# Crystal structures of (*E*)-*N*′-(2-hy­droxy-5-methyl­benzyl­idene)isonicotinohydrazide and (*E*)-*N*′-(5-fluoro-2-hy­droxy­benzyl­idene)isonicotinohydrazide

**DOI:** 10.1107/S2056989016009762

**Published:** 2016-06-17

**Authors:** Kittipong Chainok, Sureerat Makmuang, Filip Kielar

**Affiliations:** a Department of Physics, Faculty of Science and Technology, Thammasat University, Khlong Luang, Pathum Thani, 12120, Thailand; b Department of Chemistry and Center of Excellence in Biomaterials, Faculty of Science, Naresuan University, Muang, Phitsanulok, 65000, Thailand

**Keywords:** crystal structure, iron chelator, isonicotinohydrazide, hydrogen bonds

## Abstract

Two derivatives of the well-known iron chelator, (*E*)-*N*′-(2-hy­droxy­benzyl­idene)isonicotinohydrazide (SIH), substituted in the 5-position of the 2-hy­droxy­benzene ring by a methyl and a fluorine group *viz.* (*E*)-*N*′-(2-hy­droxy-5-methyl­benzyl­idene)isonicotinohydrazide, C_14_H_13_N_3_O_2_, (I), and (*E*)-*N*′-(5-fluoro-2-hy­droxy­benzyl­idene)isonicotinohydrazide, C_13_H_10_FN_3_O_2_, (II), have been prepared and characterized by single-crystal X-ray diffraction, ^1^H NMR and mass spectrometry. The mol­ecules of both compounds deviate slightly from planarity [r.m.s. deviations are 0.145 and 0.110 Å for (I) and (II), respectively] and adopt an *E* conformation with respect to the double bond of the hydrazone bridge. In each mol­ecule, there is an intra­molecular O—H⋯N hydrogen bond forming an *S*(6) ring motif. The dihedral angles between the mean planes of the isonicotinoyl ring and the cresol ring in (I) or the fluoro­phenol ring in (II) are 10.49 (6) and 9.43 (6)°, respectively. In the crystals of both compounds, zigzag chains are formed *via* N—H⋯N hydrogen bonds, in the [10-1] direction for (I) and [010] for (II). In (I), the chains are linked by weak C—H⋯π and π–π stacking inter­actions [centroid-to-centroid distances = 3.6783 (8) Å; inter-planar angle = 10.94 (5)°], leading to the formation of a three-dimensional supra­molecular architecture. In (II), adjacent chains are connected through C—H⋯O hydrogen bonds to form sheets parallel to (100), which enclose *R*_4_^4^(30) ring motifs. The sheets are linked by weak C—H⋯π and π–π [centroid-to-centroid distance = 3.7147 (8) Å; inter-planar angle = 10.94 (5)°] inter­actions, forming a three-dimensional supra­molecular architecture.

## Abstract 2

The title isonicotinohydrazides adopt an *E* conformation about the C=N bonds and in each mol­ecule there is an intra­molecular O—H⋯N hydrogen bond, forming an *S*(6) ring motif. In the crystals of both compounds, zigzag chains are formed *via* N—H⋯N hydrogen bonds, in the [10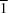] the first compound and [010] for the other.

## Chemical context   

Hydrazone-based chelators for metal ions have received a significant amount of attention (Bendova *et al.*, 2010 ▸; Hrušková *et al.*, 2016 ▸). Compounds from this class, such as salicyl aldehyde isonicotinoyl hydrazide (SIH), have been studied as potential metal chelators in biological systems (Hrušková *et al.*, 2011 ▸). These compounds have also been shown to be effective in protecting against metal-based oxidative stress (Jansová *et al.*, 2014 ▸). In our research we are inter­ested in developing probes for metal ions (Carter *et al.*, 2014 ▸). We have therefore synthesized the title compounds, which are derivatives of the chelator SIH containing a signalling unit.

## Structural commentary   

**Figure 1:**
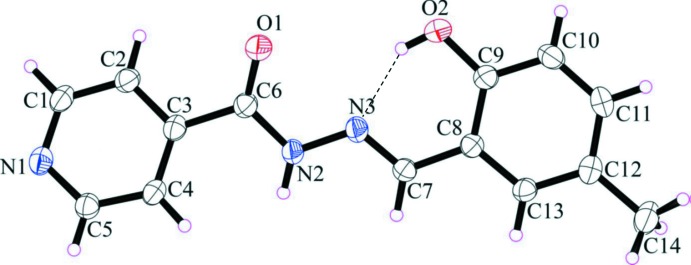
The mol­ecular structure of compound (I), showing the atom-numbering scheme. Displacement ellipsoids are drawn at the 40% probability level. The intra­molecular O—H⋯N hydrogen bond is shown as a dashed line (see Table 1 ▸).

The mol­ecular structures of the title compounds, (I) and (II), are illustrated in Figs. 1 ▸ and 2 ▸, respectively. They consist of an isonicotinoyl moiety linked by a –C7=N3–N2– linkage to a cresol unit in (I) and a fluoro­phenol ring in (II). The mol­ecules deviate slightly from planarity with the r.m.s deviations for the fitted atoms being 0.145 for (I) and 0.110 Å for (II). In each mol­ecule, there is an intra­molecular O—H⋯N hydrogen bond forming an *S*(6) ring motif. Both compounds have an *E* conformation with respect to the double bond of the hydrazone bridge (C7=N3) with the C8—C7=N3—N2 torsion angles being −179.03 (12) and −177.61 (11)° for (I) and (II), respectively. The dihedral angles between the mean planes of the isonicotinoyl moiety and the cresol moiety in (I), or the fluoro­phenol moiety in (II) are 10.49 (6) and 9.43 (6)°, respectively. The bond lengths and angles in the title mol­ecules agree reasonably well with those found in closely related structures (Chumakov *et al.*, 2001 ▸; Yang, 2006*a* ▸,*b* ▸; Kargar *et al.*, 2010 ▸; Sedaghat *et al.*, 2014 ▸)
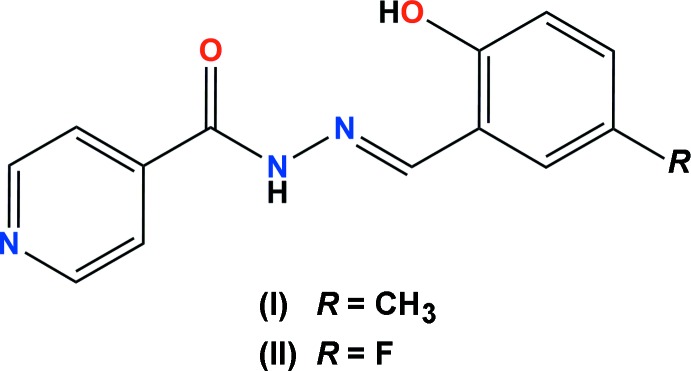
.

## Supra­molecular features   

**Figure 3:**
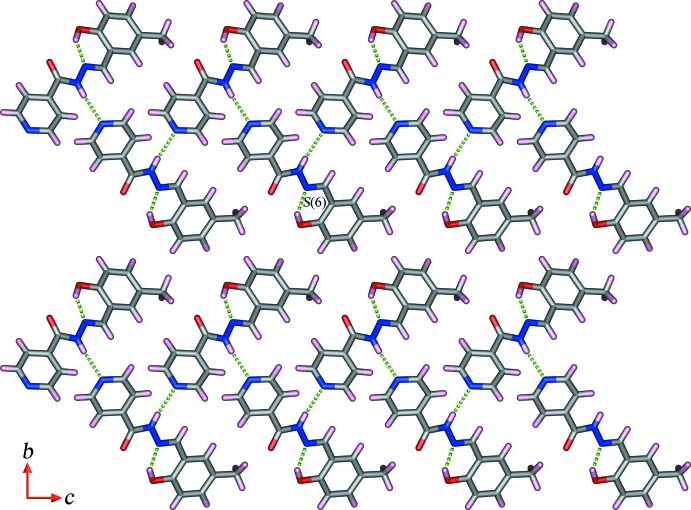
Partial view along the *a* axis of the crystal packing of compound (I), showing the hydrogen-bonded (dashed lines; see Table 1 ▸) zigzag chains parallel to [10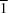].

**Figure 4:**
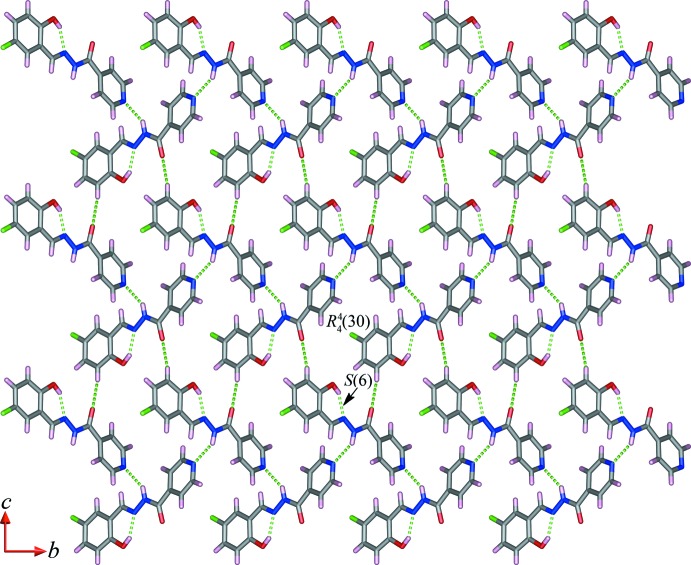
Partial view along the *a* axis of the crystal packing of compound (II), showing the N—H⋯N and C—H⋯O hydrogen-bonded (dashed lines; see Table 2 ▸) sheet propagating in the *bc* plane.

In the crystals of both compounds, zigzag chains are formed *via* N—H⋯N hydrogen bonds (Tables 1 ▸ and 2 ▸), in direction [10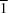] for (I) and [010] for (II). In (I), the chains are linked by weak C—H⋯π and π–π stacking inter­actions [centroid-to-centroid distances = 3.6783 (8) Å; inter-planar angle = 10.94 (5)°], leading to the formation of a three-dimensional supra­molecular architecture (Fig. 3 ▸). In (II), adjacent chains are connected through C—H⋯O hydrogen bonds to form sheets parallel to (100), which enclose 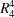
(30) ring motifs. Weak C—H⋯π and π—π [centroid-to-centroid distance = 3.7147 (8) Å, inter-planar angle = 10.94 (5)°] inter­actions link the sheets, forming a three-dimensional supra­molecular architecture (Fig. 4 ▸).

## Database survey   

A search of the Cambridge Structural Database (Version 5.37, last update November 2015; Groom *et al.*, 2016 ▸) indicated the presence of 40 structures containing the (*E*)-*N*-(2-hy­droxy­bezylydene)isonicotinohydrazide substructure. They include the isotypic crystal structures with chloride (UCAREV, Chumakov *et al.*, 2001 ▸; UCAREV01, Yang, 2006*a* ▸), bromide (XENDOK, Yang, 2006*b* ▸; XENDOK01, Sedaghat *et al.*, 2014 ▸) and meth­oxy (VACHAK, Kargar *et al.*, 2010 ▸) groups substituted at the 5-position of the phenyl ring. In the crystals of all three compounds, the N—H⋯N hydrogen bond involving the hydrazone hydrogen and the pyridine nitro­gen atoms organize the mol­ecules into a herringbone motif, while in the crystal of the meth­oxy compound there are also weak N—H⋯O and C—H⋯O hydrogen bonds present forming 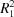
(6) ring motifs.

## Synthesis and crystallization   

A solution of isonicotinic acid hydrazide (0.184 g, 1.34 mmol) and the appropriately substituted salicyl aldehyde (1.47 mmol) in a mixture of ethanol (3 ml) and water (1 ml) containing a catalytic amount of acetic acid was heated to reflux for 5 h. The reaction mixture was allowed to cool to room temperature, resulting in the formation of a white precipitate. The reaction mixture was filtered and the isolated solid was washed with diethyl ether and dried *in vacuo*. The compounds were isolated as white crystalline solids in 73% and 66% yield for the methyl (I) and fluoro (II) derivatives, respectively. Single crystals suitable for X-ray diffraction were grown by slow evaporation of methano­lic solutions of the title compounds.

Spectroscopic data for (I): ^1^H NMR (400 MHz, DMSO-*d*_6_) *d* 2.25 (1H, *s*, CH_3_), 6.84 (1H, *d*, *J* = 8.4, CH—Ph), 7.12 (1H, *dd*, *J* = 2.0, *J* = 8.4, CH—Ph), 7.40 (1H, *d*, *J* = 1.6, CH—Ph), 7.84 (2H, *d*, *J* = 6.0, CH—Py), 8.63 (1H, *s*, CH=N), 8.79 (2H, *d*, *J* = 6.0, CH—Py), 10.82 (1H, *s*, NH), 12.26 (1H, *s*, OH). HR–MS (ES^+^) C_14_H_14_N_3_O_2_ requires 256.1086 [*M*+H]^+^; found 256.1051.

Spectroscopic data for (II): ^1^H NMR (400 MHz, DMSO-*d*_6_) d 6.94 (1H, *dd*, *J* = 4.4, *J* = 8.8, CH—Ph), 7.16 (1H, *td*, *J* = 3.2, *J* = 8.8, CH—Ph), 7.46 (1H, *dd*, *J* = 3.2, *J* = 9.6, CH—Ph), 7.84 (2H, *d*, *J* = 6.0, CH—Py), 8.67 (1H, *s*, CH=N), 8.80 (2H, *d*, *J* = 6.0, CH—Py), 10.84 (1H, *s*, NH), 12.35 (1H, *s*, OH). HR–MS (ES^+^) C_13_H_11_FN_3_O_2_ requires 260.0835 [*M*+H]^+^; found 260.0831.

## Refinement   

Crystal data, data collection and structure refinement details are summarized in Table 3 ▸. H atoms bonded to C, N, and O atoms were placed at calculated positions and refined using a riding-model approximation: N—H = 0.86 Å, O—H = 0.82 Å, and C—H = 0.93–0.96 Å with *U*_iso_(H) = 1.5*U*_eq_(C-methyl,O) and 1.2*U*_eq_(N,C) for other H atoms.

## Supplementary Material

Crystal structure: contains datablock(s) Global, I, II. DOI: 10.1107/S2056989016009762/su5301sup1.cif

Structure factors: contains datablock(s) I. DOI: 10.1107/S2056989016009762/su5301Isup2.hkl

Supporting information file. DOI: 10.1107/S2056989016009762/su5301Isup4.cdx

Structure factors: contains datablock(s) II. DOI: 10.1107/S2056989016009762/su5301IIsup3.hkl

Supporting information file. DOI: 10.1107/S2056989016009762/su5301Isup5.cml

Supporting information file. DOI: 10.1107/S2056989016009762/su5301IIsup6.cml

CCDC references: 1485834, 1485833

Additional supporting information:  crystallographic information; 3D view; checkCIF report

## Figures and Tables

**Figure 2:**
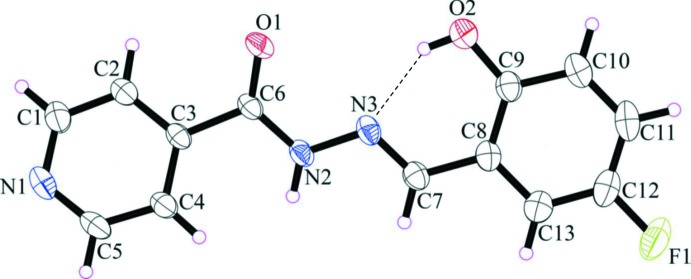
The mol­ecular structure of compound (II), showing the atom-numbering scheme. Displacement ellipsoids are drawn at the 40% probability level. The intra­molecular O—H⋯N hydrogen bond is shown as a dashed line (see Table 2 ▸).

**Table 1:** Hydrogen-bond geometry (Å, °) for (I) *Cg*1 is the centroid of the N1/C1–C5 ring.

*D*—H⋯*A*	*D*—H	H⋯*A*	*D*⋯*A*	*D*—H⋯*A*
O2—H2*O*⋯N3	0.82	1.87	2.5857 (16)	145
N2—H2*N*⋯N1^i^	0.86	2.19	3.0232 (17)	164
C10—H10⋯*Cg*1^ii^	0.93	2.85	3.5259 (17)	130

Symmetry codes: (i) 
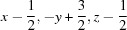
; (ii) 
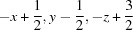
.

**Table 2:** Hydrogen-bond geometry (Å, °) for (II) *Cg*1 is the centroid of the N1/C1–C5 ring.

*D*—H⋯*A*	*D*—H	H⋯*A*	*D*⋯*A*	*D*—H⋯*A*
O2—H2⋯N3	0.82	1.92	2.6329 (15)	145
N2—H2*A*⋯N1^i^	0.86	2.19	2.8889 (15)	138
C10—H10⋯O1^ii^	0.93	2.51	3.2573 (18)	138
C11—H11⋯*Cg*1^iii^	0.93	2.98	3.8917 (18)	168

Symmetry codes: (i) 
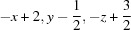
; (ii) 
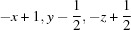
; (iii) 
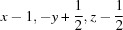
.

**Table 3:** Experimental details

	(I)	(II)
Crystal data		
Chemical formula	C_14_H_13_N_3_O_2_	C_13_H_10_FN_3_O_2_
*M*_r_	255.27	259.24
Crystal system, space group	Monoclinic, *P*2_1_/*n*	Monoclinic, *P*2_1_/*c*
Temperature (K)	296	296
*a*, *b*, *c* (Å)	8.5318 (4), 15.9973 (8), 9.4637 (5)	8.9195 (3), 10.1128 (3), 13.6254 (4)
β (°)	102.738 (2)	103.481 (1)
*V* (Å^3^)	1259.87 (11)	1195.16 (6)
*Z*	4	4
Radiation type	Mo *K*α	Mo *K*α
μ (mm^−1^)	0.09	0.11
Crystal size (mm)	0.30 × 0.22 × 0.22	0.32 × 0.26 × 0.26

Data collection		
Diffractometer	Bruker D8 QUEST CMOS	Bruker APEX2 D8 QUEST CMOS
Absorption correction	Multi-scan (*SADABS*; Bruker, 2014 ▸)	Multi-scan (*SADABS*; Bruker, 2014 ▸)
*T*_min_, *T*_max_	0.685, 0.746	0.685, 0.746
No. of measured, independent and observed [*I* > 2σ(*I*)] reflections	26052, 2996, 2111	31833, 2848, 2128
*R*_int_	0.045	0.039
(sin θ/λ)_max_ (Å^−1^)	0.659	0.658

Refinement		
*R*[*F* ^2^ > 2σ(*F* ^2^)], *wR*(*F* ^2^), *S*	0.046, 0.126, 1.01	0.042, 0.124, 1.03
No. of reflections	2996	2848
No. of parameters	174	174
H-atom treatment	H-atom parameters constrained	H-atom parameters constrained
Δρ_max_, Δρ_min_ (e Å^−3^)	0.20, −0.22	0.26, −0.29

Computer programs: *APEX2* and *SAINT* (Bruker, 2014 ▸), *SHELXT* (Sheldrick, 2015*a* ▸), *SHELXL2014* (Sheldrick, 2015*b* ▸), *OLEX2* (Dolomanov *et al.*, 2009 ▸), *ORTEP-3 for Windows* (Farrugia, 2012 ▸), *DIAMOND* (Brandenburg, 2006 ▸), *publCIF* (Westrip, 2010 ▸) and *enCIFer* (Allen *et al.*, 2004 ▸).

## supplementary crystallographic information

### (I) (E)-N'-(2-Hydroxy-5-methylbenzylidene)isonicotinohydrazide Crystal data

**Table:**

C_14_H_13_N_3_O_2_	*F*(000) = 536
*M**_r_* = 255.27	*D*_x_ = 1.346 Mg m^−^^3^
Monoclinic, *P*2_1_/*n*	Mo *K*α radiation, λ = 0.71073 Å
*a* = 8.5318 (4) Å	Cell parameters from 6456 reflections
*b* = 15.9973 (8) Å	θ = 2.9–27.3°
*c* = 9.4637 (5) Å	µ = 0.09 mm^−^^1^
β = 102.738 (2)°	*T* = 296 K
*V* = 1259.87 (11) Å^3^	Block, colourless
*Z* = 4	0.30 × 0.22 × 0.22 mm

### (I) (E)-N'-(2-Hydroxy-5-methylbenzylidene)isonicotinohydrazide Data collection

**Table:**

Bruker D8 QUEST CMOS diffractometer	2996 independent reflections
Radiation source: microfocus sealed x-ray tube, Incoatec Iµus	2111 reflections with *I* > 2σ(*I*)
GraphiteDouble Bounce Multilayer Mirror monochromator	*R*_int_ = 0.045
Detector resolution: 10.5 pixels mm^-1^	θ_max_ = 27.9°, θ_min_ = 2.9°
φ and ω scans	*h* = −11→11
Absorption correction: multi-scan (SADABS; Bruker, 2014)	*k* = −21→21
*T*_min_ = 0.685, *T*_max_ = 0.746	*l* = −12→12
26052 measured reflections	

### (I) (E)-N'-(2-Hydroxy-5-methylbenzylidene)isonicotinohydrazide Refinement

**Table:**

Refinement on *F*^2^	Primary atom site location: structure-invariant direct methods
Least-squares matrix: full	Hydrogen site location: inferred from neighbouring sites
*R*[*F*^2^ > 2σ(*F*^2^)] = 0.046	H-atom parameters constrained
*wR*(*F*^2^) = 0.126	*w* = 1/[σ^2^(*F*_o_^2^) + (0.0596*P*)^2^ + 0.2954*P*] where *P* = (*F*_o_^2^ + 2*F*_c_^2^)/3
*S* = 1.01	(Δ/σ)_max_ < 0.001
2996 reflections	Δρ_max_ = 0.20 e Å^−^^3^
174 parameters	Δρ_min_ = −0.22 e Å^−^^3^
0 restraints	

### (I) (E)-N'-(2-Hydroxy-5-methylbenzylidene)isonicotinohydrazide Special details

**Table:**

Geometry. All esds (except the esd in the dihedral angle between two l.s. planes) are estimated using the full covariance matrix. The cell esds are taken into account individually in the estimation of esds in distances, angles and torsion angles; correlations between esds in cell parameters are only used when they are defined by crystal symmetry. An approximate (isotropic) treatment of cell esds is used for estimating esds involving l.s. planes.

### (I) (E)-N'-(2-Hydroxy-5-methylbenzylidene)isonicotinohydrazide Fractional atomic coordinates and isotropic or equivalent isotropic displacement parameters (Å^2^)

**Table:**

	*x*	*y*	*z*	*U*_iso_*/*U*_eq_	
O1	0.55036 (17)	0.49610 (7)	0.82618 (13)	0.0710 (4)	
O2	0.21987 (15)	0.36743 (7)	0.62695 (11)	0.0575 (3)	
H2O	0.2779	0.4085	0.6481	0.086*	
N1	0.84669 (14)	0.76238 (8)	0.96854 (13)	0.0422 (3)	
N2	0.43283 (14)	0.57825 (7)	0.63975 (12)	0.0390 (3)	
H2N	0.4281	0.6255	0.5957	0.047*	
N3	0.33377 (14)	0.51301 (7)	0.58586 (13)	0.0392 (3)	
C1	0.81205 (18)	0.69573 (9)	1.04127 (15)	0.0430 (4)	
H1	0.8580	0.6923	1.1398	0.052*	
C2	0.71232 (18)	0.63196 (9)	0.97872 (15)	0.0418 (4)	
H2	0.6922	0.5868	1.0340	0.050*	
C3	0.64217 (16)	0.63598 (8)	0.83192 (15)	0.0362 (3)	
C4	0.67688 (16)	0.70444 (9)	0.75506 (15)	0.0377 (3)	
H4	0.6321	0.7096	0.6565	0.045*	
C5	0.77918 (17)	0.76509 (9)	0.82720 (15)	0.0406 (3)	
H5	0.8025	0.8106	0.7741	0.049*	
C6	0.53873 (18)	0.56378 (9)	0.76673 (16)	0.0415 (3)	
C7	0.23407 (16)	0.51994 (8)	0.46483 (14)	0.0365 (3)	
H7	0.2291	0.5691	0.4116	0.044*	
C8	0.12838 (16)	0.45084 (8)	0.41038 (14)	0.0336 (3)	
C9	0.12462 (17)	0.37797 (9)	0.49306 (15)	0.0393 (3)	
C10	0.02068 (19)	0.31395 (9)	0.43625 (17)	0.0463 (4)	
H10	0.0153	0.2664	0.4914	0.056*	
C11	−0.07485 (18)	0.31991 (10)	0.29884 (17)	0.0453 (4)	
H11	−0.1419	0.2755	0.2622	0.054*	
C12	−0.07365 (16)	0.39037 (10)	0.21364 (15)	0.0409 (4)	
C13	0.02749 (17)	0.45510 (9)	0.27258 (15)	0.0378 (3)	
H13	0.0283	0.5034	0.2182	0.045*	
C14	−0.1769 (2)	0.39481 (12)	0.06279 (18)	0.0601 (5)	
H14A	−0.1905	0.3397	0.0218	0.090*	
H14B	−0.1260	0.4299	0.0039	0.090*	
H14C	−0.2800	0.4176	0.0664	0.090*	

### (I) (E)-N'-(2-Hydroxy-5-methylbenzylidene)isonicotinohydrazide Atomic displacement parameters (Å^2^)

**Table:**

	*U*^11^	*U*^22^	*U*^33^	*U*^12^	*U*^13^	*U*^23^
O1	0.0903 (10)	0.0412 (7)	0.0624 (8)	−0.0114 (6)	−0.0244 (7)	0.0126 (6)
O2	0.0751 (8)	0.0487 (7)	0.0403 (6)	−0.0108 (6)	−0.0055 (5)	0.0119 (5)
N1	0.0409 (7)	0.0425 (7)	0.0401 (7)	0.0010 (5)	0.0022 (5)	−0.0074 (5)
N2	0.0408 (7)	0.0309 (6)	0.0400 (7)	−0.0020 (5)	−0.0022 (5)	−0.0019 (5)
N3	0.0409 (7)	0.0332 (6)	0.0404 (7)	−0.0023 (5)	0.0021 (5)	−0.0041 (5)
C1	0.0457 (8)	0.0465 (9)	0.0320 (7)	0.0056 (7)	−0.0018 (6)	−0.0044 (6)
C2	0.0475 (8)	0.0376 (8)	0.0368 (8)	0.0037 (6)	0.0019 (6)	0.0009 (6)
C3	0.0341 (7)	0.0348 (7)	0.0368 (7)	0.0065 (6)	0.0018 (6)	−0.0044 (6)
C4	0.0371 (8)	0.0406 (8)	0.0323 (7)	0.0043 (6)	0.0011 (6)	−0.0025 (6)
C5	0.0419 (8)	0.0392 (8)	0.0391 (8)	0.0003 (6)	0.0057 (6)	−0.0022 (6)
C6	0.0446 (8)	0.0357 (8)	0.0395 (8)	0.0018 (6)	−0.0007 (6)	−0.0010 (6)
C7	0.0411 (8)	0.0301 (7)	0.0368 (7)	0.0008 (6)	0.0053 (6)	0.0005 (6)
C8	0.0354 (7)	0.0314 (7)	0.0339 (7)	0.0027 (6)	0.0076 (5)	−0.0027 (5)
C9	0.0439 (8)	0.0386 (8)	0.0350 (7)	−0.0010 (6)	0.0076 (6)	0.0011 (6)
C10	0.0547 (9)	0.0361 (8)	0.0491 (9)	−0.0080 (7)	0.0136 (7)	0.0034 (6)
C11	0.0411 (8)	0.0418 (8)	0.0531 (9)	−0.0100 (7)	0.0107 (7)	−0.0105 (7)
C12	0.0348 (7)	0.0454 (8)	0.0406 (8)	0.0022 (6)	0.0046 (6)	−0.0080 (6)
C13	0.0408 (8)	0.0344 (7)	0.0362 (7)	0.0032 (6)	0.0041 (6)	0.0012 (6)
C14	0.0527 (10)	0.0672 (11)	0.0513 (10)	−0.0003 (9)	−0.0081 (8)	−0.0085 (8)

### (I) (E)-N'-(2-Hydroxy-5-methylbenzylidene)isonicotinohydrazide Geometric parameters (Å, º)

**Table:**

O1—C6	1.2140 (17)	C5—H5	0.9300
O2—H2O	0.8200	C7—H7	0.9300
O2—C9	1.3566 (17)	C7—C8	1.4476 (19)
N1—C1	1.3371 (19)	C8—C9	1.4083 (19)
N1—C5	1.3353 (18)	C8—C13	1.3969 (18)
N2—H2N	0.8600	C9—C10	1.383 (2)
N2—N3	1.3687 (16)	C10—H10	0.9300
N2—C6	1.3547 (17)	C10—C11	1.377 (2)
N3—C7	1.2720 (17)	C11—H11	0.9300
C1—H1	0.9300	C11—C12	1.387 (2)
C1—C2	1.376 (2)	C12—C13	1.384 (2)
C2—H2	0.9300	C12—C14	1.505 (2)
C2—C3	1.3878 (19)	C13—H13	0.9300
C3—C4	1.382 (2)	C14—H14A	0.9600
C3—C6	1.5011 (19)	C14—H14B	0.9600
C4—H4	0.9300	C14—H14C	0.9600
C4—C5	1.3808 (19)		
			
C9—O2—H2O	109.5	C8—C7—H7	120.2
C5—N1—C1	116.49 (12)	C9—C8—C7	121.54 (12)
N3—N2—H2N	122.1	C13—C8—C7	120.15 (12)
C6—N2—H2N	122.1	C13—C8—C9	118.31 (12)
C6—N2—N3	115.88 (12)	O2—C9—C8	122.64 (13)
C7—N3—N2	120.30 (12)	O2—C9—C10	118.14 (13)
N1—C1—H1	118.1	C10—C9—C8	119.21 (13)
N1—C1—C2	123.76 (13)	C9—C10—H10	119.6
C2—C1—H1	118.1	C11—C10—C9	120.72 (14)
C1—C2—H2	120.5	C11—C10—H10	119.6
C1—C2—C3	119.05 (14)	C10—C11—H11	119.1
C3—C2—H2	120.5	C10—C11—C12	121.76 (13)
C2—C3—C6	117.42 (13)	C12—C11—H11	119.1
C4—C3—C2	117.92 (13)	C11—C12—C14	120.79 (14)
C4—C3—C6	124.62 (12)	C13—C12—C11	117.23 (13)
C3—C4—H4	120.6	C13—C12—C14	121.98 (15)
C5—C4—C3	118.81 (13)	C8—C13—H13	118.6
C5—C4—H4	120.6	C12—C13—C8	122.74 (13)
N1—C5—C4	123.95 (14)	C12—C13—H13	118.6
N1—C5—H5	118.0	C12—C14—H14A	109.5
C4—C5—H5	118.0	C12—C14—H14B	109.5
O1—C6—N2	122.27 (13)	C12—C14—H14C	109.5
O1—C6—C3	121.01 (13)	H14A—C14—H14B	109.5
N2—C6—C3	116.73 (12)	H14A—C14—H14C	109.5
N3—C7—H7	120.2	H14B—C14—H14C	109.5
N3—C7—C8	119.63 (13)		
			
O2—C9—C10—C11	−177.73 (14)	C5—N1—C1—C2	−0.3 (2)
N1—C1—C2—C3	−0.2 (2)	C6—N2—N3—C7	−177.70 (13)
N2—N3—C7—C8	−179.03 (12)	C6—C3—C4—C5	−177.39 (13)
N3—N2—C6—O1	3.1 (2)	C7—C8—C9—O2	−0.7 (2)
N3—N2—C6—C3	−176.69 (12)	C7—C8—C9—C10	179.63 (13)
N3—C7—C8—C9	4.8 (2)	C7—C8—C13—C12	178.65 (13)
N3—C7—C8—C13	−174.87 (13)	C8—C7—N3—N2	−179.03 (12)
C1—N1—C5—C4	0.7 (2)	C8—C9—C10—C11	1.9 (2)
C1—C2—C3—C4	0.2 (2)	C9—C8—C13—C12	−1.0 (2)
C1—C2—C3—C6	177.94 (13)	C9—C10—C11—C12	−1.5 (2)
C2—C3—C4—C5	0.1 (2)	C10—C11—C12—C13	−0.2 (2)
C2—C3—C6—O1	−19.9 (2)	C10—C11—C12—C14	178.89 (15)
C2—C3—C6—N2	159.99 (13)	C11—C12—C13—C8	1.5 (2)
C3—C4—C5—N1	−0.6 (2)	C13—C8—C9—O2	178.91 (14)
C4—C3—C6—O1	157.69 (16)	C13—C8—C9—C10	−0.7 (2)
C4—C3—C6—N2	−22.5 (2)	C14—C12—C13—C8	−177.64 (14)

### (I) (E)-N'-(2-Hydroxy-5-methylbenzylidene)isonicotinohydrazide Hydrogen-bond geometry (Å, º)

Cg1 is the centroid of the N1/C1–C5 ring.

**Table:**

*D*—H···*A*	*D*—H	H···*A*	*D*···*A*	*D*—H···*A*
O2—H2*O*···N3	0.82	1.87	2.5857 (16)	145
N2—H2*N*···N1^i^	0.86	2.19	3.0232 (17)	164
C10—H10···*Cg*1^ii^	0.93	2.85	3.5259 (17)	130

Symmetry codes: (i) *x*−1/2, −*y*+3/2, *z*−1/2; (ii) −*x*+1/2, *y*−1/2, −*z*+3/2.

### (II) (E)-N'-(5-Fluoro-2-hydroxybenzylidene)isonicotinohydrazide Crystal data

**Table:**

C_13_H_10_FN_3_O_2_	*F*(000) = 536
*M**_r_* = 259.24	*D*_x_ = 1.441 Mg m^−^^3^
Monoclinic, *P*2_1_/*c*	Mo *K*α radiation, λ = 0.71073 Å
*a* = 8.9195 (3) Å	Cell parameters from 9934 reflections
*b* = 10.1128 (3) Å	θ = 3.1–28.5°
*c* = 13.6254 (4) Å	µ = 0.11 mm^−^^1^
β = 103.481 (1)°	*T* = 296 K
*V* = 1195.16 (6) Å^3^	Block, colourless
*Z* = 4	0.32 × 0.26 × 0.26 mm

### (II) (E)-N'-(5-Fluoro-2-hydroxybenzylidene)isonicotinohydrazide Data collection

**Table:**

Bruker APEX2 D8 QUEST CMOS diffractometer	2848 independent reflections
Radiation source: microfocus sealed x-ray tube, Incoatec Iµus	2128 reflections with *I* > 2σ(*I*)
GraphiteDouble Bounce Multilayer Mirror monochromator	*R*_int_ = 0.039
Detector resolution: 10.5 pixels mm^-1^	θ_max_ = 27.9°, θ_min_ = 3.1°
φ and ω scans	*h* = −11→11
Absorption correction: multi-scan (SADABS; Bruker, 2014)	*k* = −13→13
*T*_min_ = 0.685, *T*_max_ = 0.746	*l* = −17→17
31833 measured reflections	

### (II) (E)-N'-(5-Fluoro-2-hydroxybenzylidene)isonicotinohydrazide Refinement

**Table:**

Refinement on *F*^2^	Hydrogen site location: inferred from neighbouring sites
Least-squares matrix: full	H-atom parameters constrained
*R*[*F*^2^ > 2σ(*F*^2^)] = 0.042	*w* = 1/[σ^2^(*F*_o_^2^) + (0.0595*P*)^2^ + 0.282*P*] where *P* = (*F*_o_^2^ + 2*F*_c_^2^)/3
*wR*(*F*^2^) = 0.124	(Δ/σ)_max_ < 0.001
*S* = 1.03	Δρ_max_ = 0.26 e Å^−^^3^
2848 reflections	Δρ_min_ = −0.29 e Å^−^^3^
174 parameters	Extinction correction: SHELXL2014 (Sheldrick, 2015b), Fc^*^=kFc[1+0.001xFc^2^λ^3^/sin(2θ)]^-1/4^
0 restraints	Extinction coefficient: 0.020 (3)
Primary atom site location: structure-invariant direct methods	

### (II) (E)-N'-(5-Fluoro-2-hydroxybenzylidene)isonicotinohydrazide Special details

**Table:**

Geometry. All esds (except the esd in the dihedral angle between two l.s. planes) are estimated using the full covariance matrix. The cell esds are taken into account individually in the estimation of esds in distances, angles and torsion angles; correlations between esds in cell parameters are only used when they are defined by crystal symmetry. An approximate (isotropic) treatment of cell esds is used for estimating esds involving l.s. planes.

### (II) (E)-N'-(5-Fluoro-2-hydroxybenzylidene)isonicotinohydrazide Fractional atomic coordinates and isotropic or equivalent isotropic displacement parameters (Å^2^)

**Table:**

	*x*	*y*	*z*	*U*_iso_*/*U*_eq_	
F1	0.20524 (13)	−0.04313 (11)	0.46463 (10)	0.0868 (4)	
O1	0.82356 (13)	0.59703 (11)	0.44402 (7)	0.0572 (3)	
O2	0.53778 (15)	0.31773 (13)	0.30688 (8)	0.0650 (3)	
H2	0.5959	0.3608	0.3508	0.097*	
N1	1.11088 (14)	0.82083 (13)	0.75798 (9)	0.0497 (3)	
N2	0.75299 (13)	0.47336 (11)	0.56431 (8)	0.0404 (3)	
H2A	0.7633	0.4582	0.6276	0.048*	
N3	0.65442 (13)	0.39828 (11)	0.49277 (8)	0.0410 (3)	
C1	1.12858 (18)	0.81537 (17)	0.66408 (12)	0.0553 (4)	
H1	1.2031	0.8689	0.6466	0.066*	
C2	1.04204 (17)	0.73427 (16)	0.59110 (11)	0.0497 (4)	
H2B	1.0577	0.7346	0.5260	0.060*	
C3	0.93237 (14)	0.65288 (12)	0.61510 (9)	0.0355 (3)	
C4	0.91374 (17)	0.65656 (14)	0.71277 (10)	0.0429 (3)	
H4	0.8417	0.6026	0.7327	0.052*	
C5	1.00507 (19)	0.74261 (15)	0.78056 (10)	0.0502 (4)	
H5	0.9910	0.7455	0.8460	0.060*	
C6	0.83297 (15)	0.57142 (13)	0.53241 (9)	0.0376 (3)	
C7	0.57486 (15)	0.31041 (13)	0.52504 (10)	0.0413 (3)	
H7	0.5825	0.3020	0.5940	0.050*	
C8	0.47255 (15)	0.22351 (13)	0.45483 (10)	0.0408 (3)	
C9	0.46263 (16)	0.22688 (15)	0.35051 (11)	0.0457 (3)	
C10	0.37199 (18)	0.13466 (17)	0.28799 (13)	0.0569 (4)	
H10	0.3694	0.1346	0.2194	0.068*	
C11	0.28665 (18)	0.04408 (16)	0.32564 (14)	0.0597 (4)	
H11	0.2260	−0.0174	0.2834	0.072*	
C12	0.29224 (18)	0.04568 (15)	0.42667 (15)	0.0567 (4)	
C13	0.38397 (17)	0.13144 (15)	0.49236 (12)	0.0499 (4)	
H13	0.3870	0.1282	0.5610	0.060*	

### (II) (E)-N'-(5-Fluoro-2-hydroxybenzylidene)isonicotinohydrazide Atomic displacement parameters (Å^2^)

**Table:**

	*U*^11^	*U*^22^	*U*^33^	*U*^12^	*U*^13^	*U*^23^
F1	0.0769 (7)	0.0678 (7)	0.1118 (9)	−0.0293 (6)	0.0143 (7)	0.0139 (6)
O1	0.0767 (7)	0.0616 (7)	0.0271 (5)	−0.0163 (6)	−0.0006 (5)	−0.0004 (4)
O2	0.0697 (8)	0.0754 (8)	0.0453 (6)	−0.0252 (6)	0.0041 (5)	−0.0007 (6)
N1	0.0495 (7)	0.0494 (7)	0.0426 (7)	0.0033 (6)	−0.0048 (5)	−0.0125 (5)
N2	0.0452 (6)	0.0399 (6)	0.0297 (5)	−0.0020 (5)	−0.0040 (4)	−0.0007 (4)
N3	0.0404 (6)	0.0380 (6)	0.0379 (6)	0.0007 (5)	−0.0044 (5)	−0.0031 (5)
C1	0.0519 (9)	0.0616 (10)	0.0502 (9)	−0.0136 (8)	0.0077 (7)	−0.0123 (7)
C2	0.0530 (8)	0.0598 (9)	0.0356 (7)	−0.0099 (7)	0.0092 (6)	−0.0088 (6)
C3	0.0382 (7)	0.0351 (6)	0.0291 (6)	0.0058 (5)	−0.0007 (5)	−0.0021 (5)
C4	0.0532 (8)	0.0409 (7)	0.0324 (6)	0.0015 (6)	0.0051 (6)	−0.0022 (5)
C5	0.0662 (9)	0.0509 (8)	0.0294 (6)	0.0075 (8)	0.0026 (6)	−0.0066 (6)
C6	0.0418 (7)	0.0377 (7)	0.0285 (6)	0.0027 (6)	−0.0011 (5)	−0.0016 (5)
C7	0.0413 (7)	0.0394 (7)	0.0393 (7)	0.0042 (6)	0.0013 (6)	−0.0011 (6)
C8	0.0348 (7)	0.0358 (7)	0.0475 (7)	0.0039 (5)	0.0008 (5)	−0.0004 (6)
C9	0.0396 (7)	0.0462 (8)	0.0469 (8)	−0.0005 (6)	0.0016 (6)	−0.0020 (6)
C10	0.0514 (9)	0.0605 (10)	0.0525 (9)	−0.0037 (8)	−0.0006 (7)	−0.0119 (7)
C11	0.0464 (8)	0.0472 (9)	0.0759 (12)	−0.0042 (7)	−0.0051 (8)	−0.0132 (8)
C12	0.0435 (8)	0.0398 (8)	0.0824 (12)	−0.0038 (6)	0.0060 (8)	0.0060 (7)
C13	0.0452 (8)	0.0445 (8)	0.0572 (9)	0.0022 (6)	0.0060 (7)	0.0051 (7)

### (II) (E)-N'-(5-Fluoro-2-hydroxybenzylidene)isonicotinohydrazide Geometric parameters (Å, º)

**Table:**

F1—C12	1.3651 (19)	C3—C6	1.5061 (17)
O1—C6	1.2154 (15)	C4—H4	0.9300
O2—H2	0.8200	C4—C5	1.387 (2)
O2—C9	1.3537 (18)	C5—H5	0.9300
N1—C1	1.3263 (19)	C7—H7	0.9300
N1—C5	1.322 (2)	C7—C8	1.4537 (18)
N2—H2A	0.8600	C8—C9	1.404 (2)
N2—N3	1.3783 (15)	C8—C13	1.393 (2)
N2—C6	1.3513 (17)	C9—C10	1.388 (2)
N3—C7	1.2775 (18)	C10—H10	0.9300
C1—H1	0.9300	C10—C11	1.365 (2)
C1—C2	1.378 (2)	C11—H11	0.9300
C2—H2B	0.9300	C11—C12	1.366 (2)
C2—C3	1.375 (2)	C12—C13	1.372 (2)
C3—C4	1.3796 (18)	C13—H13	0.9300
			
C9—O2—H2	109.5	N2—C6—C3	115.07 (11)
C5—N1—C1	116.85 (12)	N3—C7—H7	119.7
N3—N2—H2A	120.8	N3—C7—C8	120.53 (13)
C6—N2—H2A	120.8	C8—C7—H7	119.7
C6—N2—N3	118.31 (11)	C9—C8—C7	122.23 (13)
C7—N3—N2	116.98 (11)	C13—C8—C7	119.03 (13)
N1—C1—H1	118.4	C13—C8—C9	118.72 (13)
N1—C1—C2	123.25 (15)	O2—C9—C8	122.70 (13)
C2—C1—H1	118.4	O2—C9—C10	117.61 (14)
C1—C2—H2B	120.2	C10—C9—C8	119.68 (14)
C3—C2—C1	119.64 (13)	C9—C10—H10	119.5
C3—C2—H2B	120.2	C11—C10—C9	121.06 (15)
C2—C3—C4	117.76 (12)	C11—C10—H10	119.5
C2—C3—C6	118.48 (11)	C10—C11—H11	120.7
C4—C3—C6	123.65 (12)	C10—C11—C12	118.57 (14)
C3—C4—H4	120.8	C12—C11—H11	120.7
C3—C4—C5	118.37 (14)	F1—C12—C11	118.92 (15)
C5—C4—H4	120.8	F1—C12—C13	118.30 (16)
N1—C5—C4	124.13 (13)	C11—C12—C13	122.77 (15)
N1—C5—H5	117.9	C8—C13—H13	120.5
C4—C5—H5	117.9	C12—C13—C8	119.09 (15)
O1—C6—N2	123.74 (12)	C12—C13—H13	120.5
O1—C6—C3	121.15 (12)		
			
F1—C12—C13—C8	−179.45 (13)	C4—C3—C6—N2	−18.13 (18)
O2—C9—C10—C11	−176.72 (15)	C5—N1—C1—C2	0.6 (2)
N1—C1—C2—C3	−0.8 (3)	C6—N2—N3—C7	−176.78 (12)
N2—N3—C7—C8	−177.61 (11)	C6—C3—C4—C5	−175.40 (12)
N3—N2—C6—O1	−0.3 (2)	C7—C8—C9—O2	−5.4 (2)
N3—N2—C6—C3	177.26 (10)	C7—C8—C9—C10	174.98 (13)
N3—C7—C8—C9	3.5 (2)	C7—C8—C13—C12	−177.30 (13)
N3—C7—C8—C13	−178.08 (12)	C8—C7—N3—N2	−177.61 (11)
C1—N1—C5—C4	0.2 (2)	C8—C9—C10—C11	2.9 (2)
C1—C2—C3—C4	0.1 (2)	C9—C8—C13—C12	1.1 (2)
C1—C2—C3—C6	176.36 (13)	C9—C10—C11—C12	−0.1 (2)
C2—C3—C4—C5	0.6 (2)	C10—C11—C12—F1	178.91 (14)
C2—C3—C6—O1	−16.5 (2)	C10—C11—C12—C13	−2.3 (2)
C2—C3—C6—N2	165.85 (12)	C11—C12—C13—C8	1.7 (2)
C3—C4—C5—N1	−0.8 (2)	C13—C8—C9—O2	176.21 (13)
C4—C3—C6—O1	159.53 (14)	C13—C8—C9—C10	−3.4 (2)

### (II) (E)-N'-(5-Fluoro-2-hydroxybenzylidene)isonicotinohydrazide Hydrogen-bond geometry (Å, º)

Cg1 is the centroid of the N1/C1–C5 ring.

**Table:**

*D*—H···*A*	*D*—H	H···*A*	*D*···*A*	*D*—H···*A*
O2—H2···N3	0.82	1.92	2.6329 (15)	145
N2—H2*A*···N1^i^	0.86	2.19	2.8889 (15)	138
C10—H10···O1^ii^	0.93	2.51	3.2573 (18)	138
C11—H11···*Cg*1^iii^	0.93	2.98	3.8917 (18)	168

Symmetry codes: (i) −*x*+2, *y*−1/2, −*z*+3/2; (ii) −*x*+1, *y*−1/2, −*z*+1/2; (iii) *x*−1, −*y*+1/2, *z*−1/2.
